# Exploiting immune cell metabolic machinery for functional HIV cure and the prevention of inflammaging

**DOI:** 10.12688/f1000research.11881.1

**Published:** 2018-01-30

**Authors:** Clovis S. Palmer, Riya Palchaudhuri, Hassan Albargy, Mohamed Abdel-Mohsen, Suzanne M. Crowe

**Affiliations:** 1 Centre for Biomedical Research, Macfarlane Burnet Institute for Medical Research and Public Health, Melbourne, VIC, Australia; 2 Department of Infectious Diseases, Monash University, Melbourne, VIC, Australia; 3 The Wistar Institute, Philadelphia , PA, USA

**Keywords:** immune cells, metabolic shift, HIV, immunometabolism

## Abstract

An emerging paradigm in immunology suggests that metabolic reprogramming and immune cell activation and functions are intricately linked. Viral infections, such as HIV infection, as well as cancer force immune cells to undergo major metabolic challenges. Cells must divert energy resources in order to mount an effective immune response. However, the fact that immune cells adopt specific metabolic programs to provide host defense against intracellular pathogens and how this metabolic shift impacts immune cell functions and the natural course of diseases have only recently been appreciated. A clearer insight into how these processes are inter-related will affect our understanding of several fundamental aspects of HIV persistence. Even in patients with long-term use of anti-retroviral therapies, HIV infection persists and continues to cause chronic immune activation and inflammation, ongoing and cumulative damage to multiple organs systems, and a reduction in life expectancy. HIV-associated fundamental changes to the metabolic machinery of the immune system can promote a state of “inflammaging”, a chronic, low-grade inflammation with specific immune changes that characterize aging, and can also contribute to the persistence of HIV in its reservoirs. In this commentary, we will bring into focus evolving concepts on how HIV modulates the metabolic machinery of immune cells in order to persist in reservoirs and how metabolic reprogramming facilitates a chronic state of inflammation that underlies the development of age-related comorbidities. We will discuss how immunometabolism is facilitating the changing paradigms in HIV cure research and outline the novel therapeutic opportunities for preventing inflammaging and premature development of age-related conditions in HIV ^+^ individuals.

## Introduction

Activation of immune cells in response to infection leads to upregulation of metabolic pathways for energy generation as well as biosynthesis to support proliferation and effector molecule production ^1, 2^. Although the impact of key metabolic pathways on immune cell development, fate, and functions is well described, only a handful of studies have focused on determining the impact of pathogens on the intrinsic immune cellular metabolic activities ^3– 5^. Even less is known about how these metabolic perturbations affect immunity and how they have impacted on the natural history of infectious diseases, including Mycobacterium tuberculosis ^6^, malaria ^7^, hepatitis B virus ^8^, and HIV ^4, 9– 12^. The emergence of the field of immunometabolism has given HIV researchers impetus to formulate novel theories and drive new research directions in order to dissect fundamental mechanisms underlying HIV-related chronic inflammation and viral persistence, challenges that have eluded scientists for decades.

## Challenges associated with HIV-associated inflammation and HIV reservoir persistence

Despite effective anti-retroviral therapy (ART), two key clinical challenges remain elusive. First, HIV remains in latent reservoirs within CD4 ^+^ T cells and possibly macrophages ^13– 16^. Thus, while effective treatment keeps immune deficiency at bay, residual HIV replication continues in tissues, including within the gut and brain. These tissue-resident reservoirs pose a major barrier for eradication and cure because once ART is interrupted, viral replication re-emerges. However, there is continuing debate regarding the contribution to the HIV reservoir of persistent viral production or the clonal expansion of immune cells containing replication-competent virus or both. Indeed, genetic characterization of HIV in HIV ^+^ART ^+^ patients has established that persistence is driven by homeostatic proliferation and clonal expansion of the active and latent HIV reservoir ^17– 19^. Under physiological conditions, memory CD4 ^+^ T cells undergo slow turnover (basal homeostatic proliferation) ^20^ but can divide rapidly in the presence of inflammatory cytokines (acute homeostatic proliferation) ^21^. Interestingly, preferential integration of HIV has been shown to occur in genes that regulate cancer and cell growth ^18^, which may influence viral transcription, cellular physiology, and metabolism. Although some studies have shown that HIV integration occurs randomly, though biased toward transcriptionally active genes, cumulative evidence supports integration site preferences by HIV ^22^.

The vast majority of efforts related to HIV cure research have focused on reactivation of latently infected cells which may then allow immune recognition and elimination by cytotoxic T cells or by cytopathic effects or both. Major caveats of this approach have recently been discussed and include the systematic release of pro-inflammatory cytokines caused by latency reversal agents (LRAs) and the problem that US Food and Drug Administration-approved LRAs re-activate only a small fraction of the latent HIV, well below the threshold required to make any appreciable and clinically significant impact on the reservoir size. Aside from LRAs’ efficiency in shocking latently infected cells and forcing them to produce virus, cytotoxic T cells are functionally exhausted in HIV-infected individuals, thus potentially dampening the capacity to clear the re-activated reservoir cells. A recent report by Borducchi *et al*. showed promising results using a therapeutic vaccination—recombinant adenovirus serotype 26 (Ad26) prime, modified vaccinia Ankara (MVA)—boosted with a non-conventional TLR7 agonist (as LRA) in decreasing HIV DNA levels and delaying viral rebound after ART interruption in a non-human primate model of SIV infection ^23^. However, more data in human clinical trials will be needed in order to validate the potential of this strategy in achieving a functional cure for HIV-1 infection. Furthermore, via *in vitro* and *ex vivo* models, it has been shown that histone deacetylase inhibitors, disulfiram, and other promising LRAs impair cytotoxic T lymphocyte (CTL) killing of HIV-infected cells ^24^, impact the function of primary natural killer (NK) cells, and have a heterogeneous mixed effect on antiviral activity, cytotoxicity, cytokine secretion, phenotype, and viability ^25^. On the other hand, recent reports have demonstrated that the effects of LRAs on anti-HIV immunity *in vitro* do not necessarily reflect with accuracy the immunologic consequences of administration *in vivo* ^24, 26^. Therefore, it will be important to comprehensively evaluate the impact of potential HIV curative strategies, especially LRAs, on anti-HIV immunity *in vivo*.

The second critical challenge in the ART era is the increased prevalence of age-related comorbidities such as cardiovascular disease, non-AIDS cancers, bone and renal disease, and frailty in virologically suppressed ART-treated persons. The pathogenesis of these non-communicable diseases is complex but is attributable to a generalized phenomenon: persistent immune activation and inflammation. While traditional risk factors similar to those in the general population contribute to their development, HIV infection significantly augments this risk, even in virologically suppressed individuals. The increasing prevalence of these comorbidities, in both developed and resource-limited countries, indicates that both traditional risks and HIV-related factors must be addressed for effective management of the aging HIV-infected population. Novel approaches to better understand the mechanisms that drive the development of these comorbidities have arisen via acknowledgment of the critical role of monocyte and macrophage metabolism in controlling inflammation and non-communicable disease development ^27– 32^.

This commentary will discuss a new and arguably controversial HIV cure strategy—“block and lock”—used to suppress reservoir activation ^33^, an emerging paradigm supported by new insights of metabolic regulation of latency. In addition, we will discuss targeting of host immune cellular machinery as an approach to circumvent the threat of HIV resistance against current treatments as well as to control chronic HIV-associated chronic inflammation and the related development of non-communicable comorbidities.

## Metabolic pathways regulating T-cell and monocyte metabolism

Immunometabolism, the study of the interplay between bioenergetic pathways and immune cell functions, is increasingly gaining acknowledgement as an emerging scientific field. In response to infection, activation of the host innate and adaptive immune cells is accompanied by dramatic changes in cellular bioenergetics. Resting cells predominantly derive their energy via oxidative phosphorylation (OXphos) and shift to glycolysis—a metabolic remodeling called the Warburg effect—for cell growth, proliferation, and synthesis of antimicrobial and pro-inflammatory effector molecules. A better understanding of the dynamics of these metabolic states and their specific functions during viral pathogenesis can lead to the development of immunotherapies capable of suppressing viral replication and combat the deleterious effects of persistent high-grade inflammation to hosts ^34, 35^.

All immune cells use glucose in order to produce energy to mount an effective immune response against pathogens. Glucose is metabolized via two major pathways: OXPHOS, which takes place in the mitochondria in order to produce the maximal amount of ATP, and glycolysis, which occurs in the cytosol and produces less ATP. Intermediates of glycolysis may be used as precursors for protein, lipid, and nucleotide synthesis which are needed by rapidly proliferating cells ^36^. Metabolites of cellular metabolism may also regulate several significant biological processes, such as epigenetic reprogramming, which influences cytokine production by immune cells such as macrophages ^37^. Furthermore, glycolytic enzymes such as GAPDH can bind to the adenylate uridylate (AU)-rich elements within the 3′ untranslated region (3′ UTR) of mRNA that encodes cytokines such as interferon-gamma (IFN-γ). Thus, by engaging or disengaging glycolysis through changes in its expression, GAPDH may control inflammatory and effector responses ^38^. Aerobic glycolysis can also control IFN-γ expression independently of the 3′ UTR through increased activity of lactate dehydrogenase by maintaining high concentrations of acetyl-coenzyme A (acetyl-CoA), which enhances histone acetylation and *Ifng* transcription ^39^.

More recently, there has been an appreciation of the differential use of glucose and fatty acids by immune cells on the basis of their differentiation and activation status. This has been elegantly reviewed by Shehata *et al*. ^34^, who highlighted that memory T cells predominately use glucose to fuel fatty acid synthesis (FAS), which is stored and later metabolized via OXPHOS to produce ATP. This deviates from that of effector T cells where glucose is predominantly metabolized via aerobic glycolysis to produce lactate.

Thus, although this commentary focusses on the role of glucose metabolism in supporting HIV reservoir persistence and inflammation, other fundamental metabolic processes involving fatty acids ^40^ and amino acids ^41^ may play complementary roles. Indeed, glutamine uptake is increased in activated T cells to generate α-ketoglutarate via glutaminolysis to support the citric acid cycle when pyruvate and acetyl-CoA are limited ^34, 42^.

## Metabolic reprogramming of functional T-cell subsets

The activation of naïve CD4 ^+^ T cells requires metabolic reprogramming characterized by increased glycolysis and downregulation of lipid oxidation ^2, 43– 45^. These activated T cells can then differentiate into a variety of effector subsets, depending on the cytokine profile in the microenvironment ^46^. Effector, memory, and regulatory CD4 ^+^ T cells exhibit a specific metabolic phenotype, suggesting that metabolism is tightly linked to their functions ^47^. Furthermore, recent studies have identified significant metabolic flexibility between the T helper 17 (Th17) and regulatory T (Treg) cell compartments ^48, 49^. Th17 and Treg cells represent two arms of an immune response, and their uniquely plastic relationship conditions the immune environment to shift between pro- and anti-inflammatory states ^50^. With a mouse model, it was shown that Th17 cells could transdifferentiate into Treg cells ^51^.

It is becoming increasingly clear that metabolism plays a significant role in driving these distinct differentiation programs. For example, the pro-inflammatory CD4 ^+^ Th1, Th2, and Th17 lineages express high surface levels of glucose transporter 1 (Glut1) and were highly glycolytic. Contrary to this, Treg cells require oxidative metabolism to fuel suppressive functions and preferentially oxidize lipids which are AMP kinase dependent ^2, 52, 53^. It is worth noting that blocking glycolysis during *in vitro* Th17 differentiation favors Treg cell formation rather than that of Th17 cells. Furthermore, dichloroacetate (DCA), a promising immunosuppressive drug for the treatment of lactic acidosis, inhibits aerobic glycolysis in alloreactive CD4 ^+^ T cells, which favors differentiation toward Treg cells ^54^.

The metabolic transcription factor hypoxia-inducible factor 1 alpha (HIF-1α) seems to play a special role, particularly for Th17 cells. HIF-1α is a transcription factor regulating the expression of metabolic enzymes and an essential facilitator of the acquisition of Th17 glycolytic metabolism ^55^. HIF-1α deletion under Th17-promoting conditions results in a blunted upregulation of Glut1 and the reduced expression of crucial glycolytic enzymes such as hexokinase 2, glucose-6-phosphate isomerase, and lactate dehydrogenase as well as the generation of Th17 cells both *in vitro* and in Th17-promoting disease models *in vivo* ^56^.

Pyruvate dehydrogenase (PDH) is another important checkpoint for Th17 cell- or Treg cell-specific metabolic pathway decisions ^57^. The conversion of cytosolic pyruvate into mitochondrial acetyl-CoA for oxidative metabolism is catalyzed by PDH and is inhibited by PDH kinase (PDHK). PDHK is regulated by hypoxia and HIF-1α and promotes the generation of lactate by suppressing pyruvate oxidation ^58^. Through a detailed metabolic analysis, Gerriets *et al*. identified that an isoform of PDHK, PDHK1, is predominantly expressed in Th17 cells and at low levels in Treg cells but not in Th1 cells ^57^. The inhibition of PDHK1 by DCA can suppress glycolysis and selectively suppress Th17 generation ^57^.

*De novo* FAS was also shown to be essential for the generation of Th17 cells in contrast to Treg cells. Accordingly, the inhibition or deletion of acetyl-CoA carboxylase 1 (ACC1), a key enzyme for *de novo* FAS, resulted in an impaired Th17 differentiation, whereas Treg cells were induced. Moreover, blocking ACC1 activity induced a shift from Th17 toward Treg cell induction under Th17 culture conditions, together suggesting that engagement of FAS is indispensable for Th17 but not Treg cell induction ^59^. Data are lacking regarding the impact of HIV infection on the modulation of metabolism in these functional T-cell subsets and how this may influence HIV pathogenesis. The diverse players involved in metabolic programming of these subsets offer tremendous therapeutic targets that could alter HIV disease outcomes. Yet they highlight a fine balance between conflicting arms of the immune system that must be considered when administering metabolic-modulating drugs.

## HIV disrupts glucose metabolism in immune cells

Immune cells rely on OXPHOS for efficient glucose metabolism in order to generate ATP for survival and function. Early work by Sorbara *et al*. has shown that HIV-1-infected H9 T-cell lines exhibit a 10-fold increase in the expression of Glut1 within 4 days of inoculation ^60^, and replication of HIV-1 in primary CD4 ^+^ T cells increases glycolytic flux ^11^. Interestingly, Trautmann *et al*. have demonstrated that the susceptibility of HIV-specific CD8 ^+^ T cells to apoptosis in early HIV infection was associated with a metabolic state in which these cells exhaust their mitochondria to sustain their proliferation ^61^. Although functional metabolic assessment was beyond the scope of their work, it appeared that apoptosis was PI3Kinase–Akt dependent.

Work from our group has shown that CD4 ^+^ T cells and monocytes obtained from HIV ^+^ treatment-naïve and ART-treated patients exhibit a glycolytic phenotype ^4, 9, 62^ essential for the maintenance of CD4 ^+^ T-cell activation ^63^ and monocyte/macrophage pro-inflammatory cytokine production ^64^. Cellular immune activation in response to infections results in the trafficking of Glut1, the major glucose transporter in immune cells, to the cell surface as the first and major committed step in glucose metabolism. This is followed by increased glucose uptake and elevated lactate production, a glycolysis signature, shared by cancer cells in which aerobic glycolysis predominates even in the presence of sufficient oxygen, called the Warburg effect. This metabolic reprogramming is coordinated by the mechanistic target of rapamycin (mTOR), a critical regulatory kinase that serves as a key checkpoint and therapeutic target for several cancers and inflammatory and autoimmune disorders ^65, 66^.

## Intracellular pathogens have a sweet tooth for survival

Metabolic re-programming is not exclusive to cancers or HIV infection. Other intracellular pathogens exploit host metabolic machinery to avoid immune surveillance. For example, the Zika virus, associated with human congenital fetal anomalies, diverts host cell resources and reprograms metabolic processes to support RNA metabolism, ATP production, and glycolysis. This metabolic state is conducive for Zika virus replication ^67^ and may control access to nutrients (for example, glucose), a requisite for endothelial growth within the placenta and fetal development ^68^. Non-viral pathogens such as *Plasmodium berghei*, a malarial parasite of rodents, augment glucose uptake via Glut1 activity and surface localization in infected hepatic cells as an adaptive response for survival ^69^. A comparable dynamic interplay between immune cell metabolism and HIV persistence and inflammatory responses has drawn intense attention lately and will be the focus of the remaining discussion ^34, 70^.

## Mining the host metabolic machinery for a new potential HIV drug class

The mTOR is an important regulator of glucose metabolism and connects cell growth, energy balance, and aging to metabolic processes. This serine/threonine kinase comprises two distinct complexes: mTORC1, which is highly sensitive to the immunosuppressant agent rapamycin, and mTORC2, which is sensitive to rapamycin only when chronically exposed. Dual suppression of these two complexes by the mTOR inhibitor INK128 suppresses HIV entry and transcription *in vitro* and inhibits multidrug-resistant HIV in preclinical models ^71^. This observation opens up opportunities for the design of new combinatorial therapies to treat HIV-infected persons who have failed currently available ART regimens. However, concerns have been raised regarding potentially undesirable consequences of metabolic inhibitors on CD8 ^+^ T-cell functions and facilitating memory CD4 ^+^ T-cell formation and hypothetically increasing reservoir size (Steven Deeks, personal communications). Recently, these fears have been partially counteracted by evidence showing that metabolic inhibitors targeting mTOR pathways may have favorable immunomodulatory effects, including enhanced antiviral responses ^72^. Furthermore, mTOR inhibitors have been shown to facilitate memory CD8 ^+^ T-cell formation *in vivo* using murine and non-human primate models and exhibit unique differences in metabolic machinery between CD4 ^+^ and CD8 ^+^ T cells ^73^. Thereby, mTOR inhibitors are gaining increasing interest. However, their full impact on the formation of memory CD4 ^+^ T-cell subsets carrying replication-competent virus needs to be carefully evaluated *in vivo*.

## Metabolic inhibition to “block and lock” the HIV reservoir

The setbacks from clinical trials employing the “kick and kill” approach to eradicate HIV ^74, 75^ have forced researchers to re-think and explore alternative cure strategies, including the provocative “block and lock” ^76^ and “starve” ^36, 77, 78^ approaches. These methods attempt to control viral persistence by metabolic suppression of cells harboring HIV proviral DNA to subdue reactivation and homeostatic proliferation of reservoir cells. Recent data by Heredia *et al*. have brought support to this shifting chain of thought by demonstrating that HIV hyperactivates mTORC1 activity in a PI3Kinase-dependent manner promoting the synthesis of biomolecules for virion production and latent viral reactivation ^71^. Furthermore, inhibition of mTORC1 or PI3Kinase can successfully inhibit viral replication and viral reactivation as a result of a decrease in cellular biosynthesis ^3, 79^. A confirmatory role of the involvement of mTOR in controlling HIV latency was also established by employing a genome-wide screen approach by Verdin *et al*., who used different HIV latency models and HIV-infected patient primary cells ^76^. They have shown that the dual mTOR inhibitors Torin1 and pp242, which both target the two mTOR complexes (mRORC1 and mTORC2), strongly suppressed latent HIV reactivation following potent CD4 T-cell activation through the T-cell receptor ^76^. Furthermore, inhibition of these complexes abrogated both Tat-dependent and Tat-independent transactivation of the HIV promotor ^76^. Thus, whereas the “block and lock” predominantly focusses on limiting the transcription of latent HIV DNA through mTOR suppression, the “starve” model earlier proposed by Palmer *et al*. posits subduing the metabolic activity of reservoir cells to limit homeostatic proliferation while controlling the metabolic state within T cells and macrophages, essential for viral infectivity ^36, 78^.

## mTOR and latency-reversing agents: toward an HIV cure

Recent work by Siliciano’s group has provided an experimental model supporting an interesting combination approach by which mTOR drugs could mitigate LRA-mediated inflammatory responses and toxicity while maximally reactivating the HIV reservoir ^80^. In their *in vitro* latency model, rapamycin did not inhibit HIV reactivation in a dose-dependent manner in activated CD4 ^+^ T cells from individuals on suppressive ART. These results are compatible with the finding that rapamycin inhibits mTORC1 but not mTORC2. The action of rapamycin on mTORC1 causes repression of the basal transcription of HIV long-terminal repeat (LTR) without affecting Tat-mediated transactivation of the virus ^81^. Inhibition of mTORC2, on the other hand, appears to inhibit both basal transcription and tat-mediated HIV transcription ^71^. However, rapamycin drastically reduced the secretion of pro-inflammatory cytokines and suppressed the proliferation of CD4 ^+^ T cells from these patients. Furthermore, rapamycin reduced the expression of the T-cell exhaustion marker PD-1 while preserving basal CTL-mediated killing of infected cells ^80^. However, other pro-inflammatory factors produced by activated CD4 ^+^ T cells (for example, inflammatory lipids such as ceramides) may result in clinically deleterious inflammatory responses that may not be diminished by mTOR inhibitors. Nonetheless, the observational study by Stock *et al*., in which rapamycin treatment of HIV ^+^ kidney transplant recipients caused reduced frequency of T cells harboring HIV DNA, will likely encourage clinical trials aimed at specifically examining the effects of mTOR inhibitors and their impact on the HIV reservoir size ^82^.

Importantly, the impact of these and other glucose metabolic inhibitors (for example, those targeting specific PI3Kinase isoforms, Glut1, and hexokinase II) on potential long-term remission in ART-treated virologically suppressed HIV ^+^ individuals should be carefully evaluated. In addition, it is likely that a multidisciplinary approach that includes a combination of therapies will be necessary to achieve HIV eradication or ART-free sustained HIV control. Recently, the success of immunotherapy targeting the inhibitory receptors PD-1, CTLA-4, and other immune-negative checkpoints in recovering T-cell immunity has promoted interest in using similar strategies to achieve HIV eradication.

T-cell exhaustion is a process that depends on metabolic changes driven by signaling through these negative immune checkpoints ^83^; it has been shown that blocking immune checkpoints has differential effects on cell metabolism, depending on which molecule is targeted by the blockade ^84, 85^. Therefore, evaluation of the impact of immune checkpoint blockade therapy on cellular immunometabolism machinery may have relevance in the setting of HIV. Furthermore, combining immunotherapy and metabolism-based therapies can be another potential targeted strategy and warrants further investigation.

## Metabolic rewiring of monocytes/macrophages primes host inflammatory and defense mechanisms

Like activated T cells, Glut1 is the main inflammatory-responsive glucose transporter on activated monocytes and allows high glucose uptake required to fuel glycolysis. We have shown that, in ART-treated HIV-infected patients, Glut1 is profoundly increased on pro-inflammatory monocytes ^9^. Also, Freemerman *et al*. have found that inflammatory M1 macrophages have overwhelmingly high glycolytic activity and Glut1 expression via *in vitro* experiments using murine macrophages ^64^.

However, cytokine production by M2 polarized human macrophages has also been shown to rely on glycolytic metabolism in addition to fatty acid oxidation (FAO). Control of this glycolytic state provides the metabolic basis by which interleukin-10 (IL-10) exerts its anti-inflammatory effects. For example, IL-10 opposes lipopolysaccharide (LPS)-induced glucose uptake and glycolysis in macrophages and promotes OXPHOS in an mTOR-dependent manner ^30^. However, it should be noted that, unlike LPS alone, complex microbial stimuli can induce specific metabolic reprogramming that involves upregulation of OXPHOS, glycolysis, and FAO to prime host defense mechanisms, including cytokine production and phagocytosis.

## Immunometabolism offers new opportunities to control inflammation and age-related comorbidities in ART-treated HIV ^+^ individuals

In our earlier work, we indicated that cell-surface Glut1 levels on pro-inflammatory monocytes (the intermediate CD16 ^+^ monocyte subset) correlated significantly with inflammatory plasma biomarkers of inflammation and cardiovascular disease risk ^9^. Furthermore, Glut1-expressing monocytes exhibit higher levels of intracellular tumor necrosis factor (TNF) compared with Glut1-negative cells ^9^. However, although several models have been proposed to connect monocyte glucose metabolic dysfunction with age-related comorbidities ^36, 86^, experimental evidence has only recently come together to support these models.

Analysis of monocytes from HIV-infected participants enrolled in the Women’s Interagency HIV Study showed that the frequency of circulating Glut1-expressing intermediate monocytes is significantly elevated in those with subclinical cardiovascular disease ^10^. Furthermore, in a group of aging HIV ^+^ men on suppressive combined ART, frailty (evaluated using the Frailty Index) was associated with Glut1 expression on total monocytes ^87^. Since monocyte metabolic activation may contribute to the development of age-related comorbidities such as atherosclerosis in ART-treated HIV ^+^ persons, Glut1 is potentially a novel target to limit inflammation. One may argue against the use of metabolic inhibitors as potential immunomodulators in the context of inflammatory diseases such as HIV infection in fear of deleterious side effects. However, it is worth noting that glucose uptake by insulin-sensitive cells such as adipocytes and myocytes is controlled by other glucose transporter isoforms (for example, Glut4), which are less responsive to inflammatory signals compared with Glut1 ^88^. Furthermore, specific PI3Kinase isoforms such as PI3Kγ and PI3Kδ which control mTOR and glucose metabolism are restricted to immune cells, thereby circumventing or limiting potential side effects. The unwavering interest in immunometabolism by biotechnology companies and the current intersection between immune cell metabolism and the cancer field now provide great opportunities for re-purposing some cancer drugs to treat pathogen-driven inflammatory and non-communicable diseases ^36, 89^.

## Key points and conclusion

**Figure 1.**
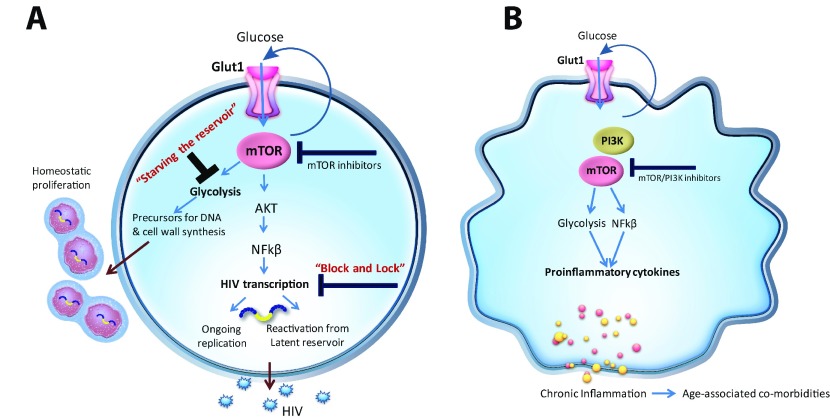
CD4 ^+^ T cells reprogram glucose metabolism during infection from oxidative phosphorylation (OXphos) toward glycolysis marked by increased cell surface Glut1 and mTOR activation. ( **a**) mTOR regulates HIV transcription critical for viral reactivation and ongoing replication. Glycolysis regulated by mTOR provides precursors for DNA and cell wall synthesis which support homeostatic proliferation of infected CD4 ^+^ T cells. ( **b**) Similar to CD4 ^+^ T cells, mTOR/PI3Kinase regulates monocyte/macrophage Glut1 cell surface expression and metabolism. A metabolic shift toward glycolysis supports pro-inflammatory cytokine production in monocytes/macrophages. Glut1, glucose transporter 1; mTOR, mechanistic target of rapamycin.

Lack of knowledge regarding the precise mechanisms underlying viral persistence and chronic inflammation in HIV infection has hampered the development of host-directed therapies to eradicate the HIV reservoir and control chronic inflammation in HIV-infected persons. Recent work has exposed previously unrecognized alterations in cellular energy metabolism in immune cells in HIV ^+^ individuals. In addition, the critical role of glucose metabolism, regulated by Glut1 and mTOR, in controlling HIV replication, latency, and inflammatory responses has been established ( Figure 1).

More studies evaluating different classes of glucose metabolic inhibitors directed against immune cells are warranted to justify their use in HIV-infected individuals. Furthermore, modulating glucose metabolic activities could be either beneficial or detrimental depending on the infection stage in which they are administered ^34, 90^. It will also be important to delineate the additional pathways that control glucose metabolism, including mitochondrial biogenesis, as additional therapeutic target options. The main challenge in identifying new metabolic networks will be the adoption of new technologies to interrogate metabolism in small blood samples typically available from clinical testing. New technologies such as the Seahorse extracellular flux analyzer have helped to revolutionize the field; however, the readout gives only a global snapshot of oxidative and glycolytic metabolism. In order to fully address the complex questions of how metabolic remodeling of immune cells contributes to the course of HIV infection, additional technologies, such as those employing multiparametric techniques to study specific subpopulations of immune cells (reviewed in 28), are needed.
